# Comment on “Refining risk stratification in hepatocellular carcinoma by integrating tertiary lymphoid structures and microvascular invasion*”*

**DOI:** 10.1097/JS9.0000000000003184

**Published:** 2025-08-06

**Authors:** Liang Chen, Jiacheng Chen

**Affiliations:** Department of Hepatobiliary & Pancreatic Surgery, Hainan General Hospital (Hainan Affiliated Hospital of Hainan Medical University), Haikou, Hainan Province China

*Dear Editor,*

We read with great interest the article titled *“Refining Risk Stratification in Hepatocellular Carcinoma by Integrating Tertiary Lymphoid Structures and Microvascular Invasion*^[1]^*”*. This multicenter retrospective study contributes valuable insights into histopathological risk stratification in hepatocellular carcinoma (HCC), and we commend the authors for their rigorous design and novel integration of TLS and MVI as prognostic markers.This correspondence was developed following the TITAN Guidelines 2025^[2]^ for the declaration and use of artificial intelligence in scholarly publishing. No AI tools were employed in the conception, design, analysis, interpretation, or writing of this work.

However, we wish to respectfully highlight a potential methodological concern . The authors evaluated TLS status solely based on hematoxylin and eosin (H&E) staining, without incorporation of immunohistochemical (IHC) markers or spatial structure-based scoring systems. While the reported interobserver agreement was substantial (Kappa = 0.79), reliance on H&E morphology alone may still introduce interpretative variability, especially when differentiating between TLS aggregates and reactive lymphoid infiltrates in cirrhotic or inflamed liver tissue.

Increasing evidence supports the use of multi-marker IHC panels (e.g., CD3, CD20, PNAd, and CD21) or spatial transcriptomic profiling to objectively identify and classify TLS, enabling distinction between mature, functional TLS, and immature or nonspecific lymphoid clusters^[3,4]^. Such approaches have been shown to enhance reproducibility and biological relevance, particularly when TLS status is used to inform immunological or therapeutic stratification in clinical research.

Given that TLS status served as a key grouping factor influencing recurrence-free survival and treatment responsiveness in this study, we believe that a more standardized and objective TLS definition should be considered in future validations to strengthen clinical applicability.

## Data Availability

The letter does not have any data.

## Research registration unique identifying number (UIN)

Not registered for the type of the study.
